# Vortex Circular Dichroism: An experimental technique to assess the scalar/vectorial regime of diffraction

**DOI:** 10.12688/openreseurope.14916.1

**Published:** 2022-12-19

**Authors:** Xavier Zambrana-Puyalto, Francesco De Angelis, Vincenzo D'Ambrosio

**Affiliations:** 1 Istituto Italiano di Tecnologia, Via Morego 30, 16163 Genova, Italy; 2 Dipartimento di Fisica, Università di Napoli Federico II, Complesso Universitario di Monte S. Angelo, Via Cintia, 80126 Napoli, Italy

**Keywords:** Angular momentum, Vortex beam, Circular Dichroism, Helicity, Non-paraxial, Mie Theory, q-plate, Nanoholes

## Abstract

**Background:** In classical electrodynamics, light-matter interactions are modeled using Maxwell equations. The solution of Maxwell equations, which is typically given by means of the electric and magnetic field, is vectorial in nature. Yet it is well known that light-matter interactions can be approximately described in a scalar (polarization-independent) way for many optical applications. While the accuracy of the scalar approximation can be theoretically computed, to the best of our knowledge, it has never been determined experimentally. Here, we show that the vectoriality of diffraction can be probed with a new technique: Vortex Circular Dichroism (VCD).

**Methods:** We measure the differential transmission of left and right circularly polarized vortex beams through a set of single circular nano-apertures with diameters ranging from 150 to 1950 nm. We observe that VCD > 0 for smaller diameters, VCD ≲ 0 for intermediate values and VCD ≈ 0 for larger values of the diameter.

We also carry out Mie Theory simulations for spheres with the same diameters as the nanoholes and observe that the theoretical and experimental VCD values follow the same trend line.

**Results:**We relate VCD ≠ 0 to a vectorial diffraction, and VCD ≈ 0 to a scalar one. This is corroborated by the simulations, which show that a diffraction process characterized by a VCD ≠ 0 (VCD ≠ 0) is polarization-independent (polarization-dependent).

**Conclusions:**Overall, our results give a wealth of evidence that VCD allows for the experimental assessment of the scalar/vectorial regime of diffraction.

## Introduction

Light is an electromagnetic (EM) field that oscillates in the 1 THz − 30 PHz band (or 10 nm −300 *µ*m in wavelength units) ^1^. For classical phenomena, the propagation of light as well as its interaction with matter is described by Maxwell equations, which are typically expressed as four differential equations involving the electric and magnetic vector fields, { **E**, **B**} ^2^. The six scalar functions { *E*_1,2,3_, *B*_1,2,3_} that make up the EM field are not independent, as they are bonded to each other via Maxwell equations. That is, given a certain *E*_1_ ( **r**, *t*), the other two components *E*_2,3_ ( **r**, *t*) must have a certain functional form. Simply put, the spatial properties of light or the three intensity patterns given by *E _i_* ( **r**, *t*) cannot be independently modified. Or in other words, the polarization (or vectorial properties) of light cannot be modified without changing its spatial properties.

**Figure 1.**
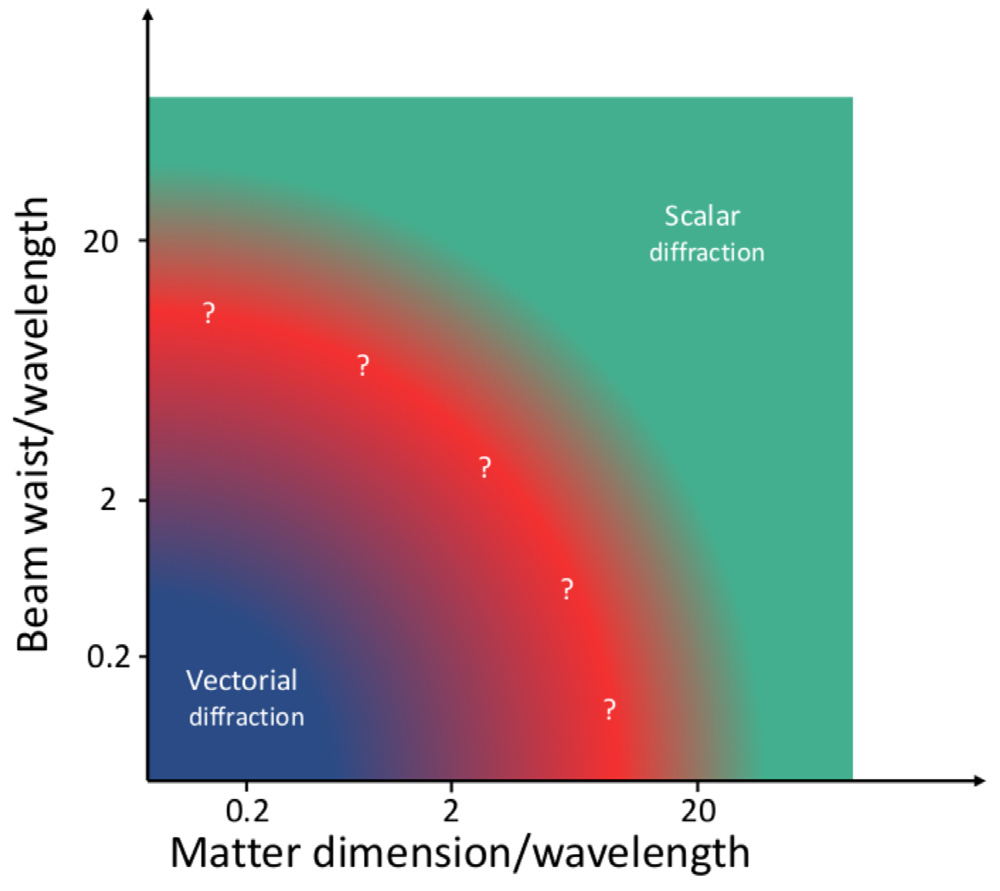
Sketch of the validity of the scalar diffraction approximation as a function of the beam waist, wavelength and matter dimensions. The image is only an indicative sketch, no calculations have been made to trace the limits between the different regions. Note that the vectorial diffraction theory is always valid, including the region where the scalar approximation can be used.

In optics, many phenomena are properly described using the wave optics approximation, *e.g.* interference ^1^, scalar diffraction theory ^3^ or holography ^4^. In such approximation, the EM field of light is described as a scalar field times a polarization vector: **E**( **r**, *t*) = *E* ( **r**, *t*) **u**, with **u** being a unitary polarization vector. As a result, the spatial properties of the field (described by the scalar function *E* ( **r**, *t*)) and its polarization **u** can be independently modified. In particular, when a beam of light is described using wave optics, we typically say that we are in the paraxial approximation ^5^. In contrast, we say that a beam is non-paraxial when its description requires a vectorial formulation ^6^. Note that when it comes to light diffraction by matter, the description regime of this interaction does not necessarily need to be the same one as the beam. For example, the diffraction of a paraxial beam by a subwavelength spherical particle generally calls for a vectorial description, which is known as Mie Theory ^7, 8^. In the same way, the far-field diffraction of a non-paraxial beam off a macroscopic mirror could be described using scalar diffraction theory. Obviously, using the scalar (wave optics) approximation makes calculations as well as the interpretations of results much simpler, yet we need to make sure that we are under the right conditions to apply it. On the one hand, the scalar regime is suitable to describe diffraction processes where the dimensions of both the beams and matter are much greater than the wavelength. On the other hand, a vectorial description is needed when the dimensions of both matter and light beams are smaller than the wavelength. In contrast, the intermediate cases (which are common in nano-optics) are much more subtle, and it is difficult to know *a priori* if the scalar approximation can properly describe the interaction. This is schematically displayed in Figure 1, where we show the regimes of beam waist, wavelength and the matter dimension that roughly define the scalar approximation, the intermediate cases, and the purely vectorial diffraction regime.

Figuring out the diffraction regime has no secrets from a theoretical perspective, as the brute force approach could always be used. That is, any diffraction process could be modeled with both Maxwell equations and wave optics, and the results could be compared. If the difference between the diffracted EM fields computed in both ways was negligible for all points in space and time, we would conclude that the system behaves in a scalar way. Following the same logic, if the difference was not negligible, we would say that the diffraction process is vectorial. The rationale that is implicit in these two statements is that the difference between the EM field (in all points of space and time) computed using Maxwell equations and a wave optics approximation is a good *measure of vectoriality*. Obviously, this measure of vectoriality precludes its experimental realization, as it is impossible to measure the EM field in all points of space and time. In this work, we propose an experimental technique that can be used to define a measure of vectoriality which is zero when the diffraction process is scalar and different from zero when it is vectorial. We call this experimental technique Vortex Circular Dichroism (VCD).

VCD is a technique based on a Circular Dichorism (CD) measurement with beams that carry a phase singularity, *i.e.* vortex beams ^9^. The CD measurement is obtained as the differential absorption of the left and right circularly polarized (LCP and RCP) components of a light beam. CD is widely used in molecular and protein spectroscopy ^10^, among others. One of its most typical uses is the study of chiral properties of molecules ^10^. It is generally accepted that the CD signal of a non-chiral object is zero, while the CD signal of a chiral object is different from zero. However, in recent years it has been demonstrated that vortex beams can induce a CD signal different from zero on non-chiral samples ^11^. In this work, it was shown that the main difference between a standard CD measurement using Gaussian beams or plane waves, and the CD measurement using vortex beams is that in the latter the two LCP and RCP beams that are subtracted have different angular momentum content. Here, we shall prove that the underlying reason why a CD measurement using vortex beams ( *i.e.* VCD) can yield a result different from zero with a non-chiral sample is the fact that the interaction between the vortex beam and the sample is vectorial. That is, we will show that VCD is a technique that allows us to define a measure of vectoriality and therefore assess if a diffraction process is scalar or vectorial.

**Figure 2.**
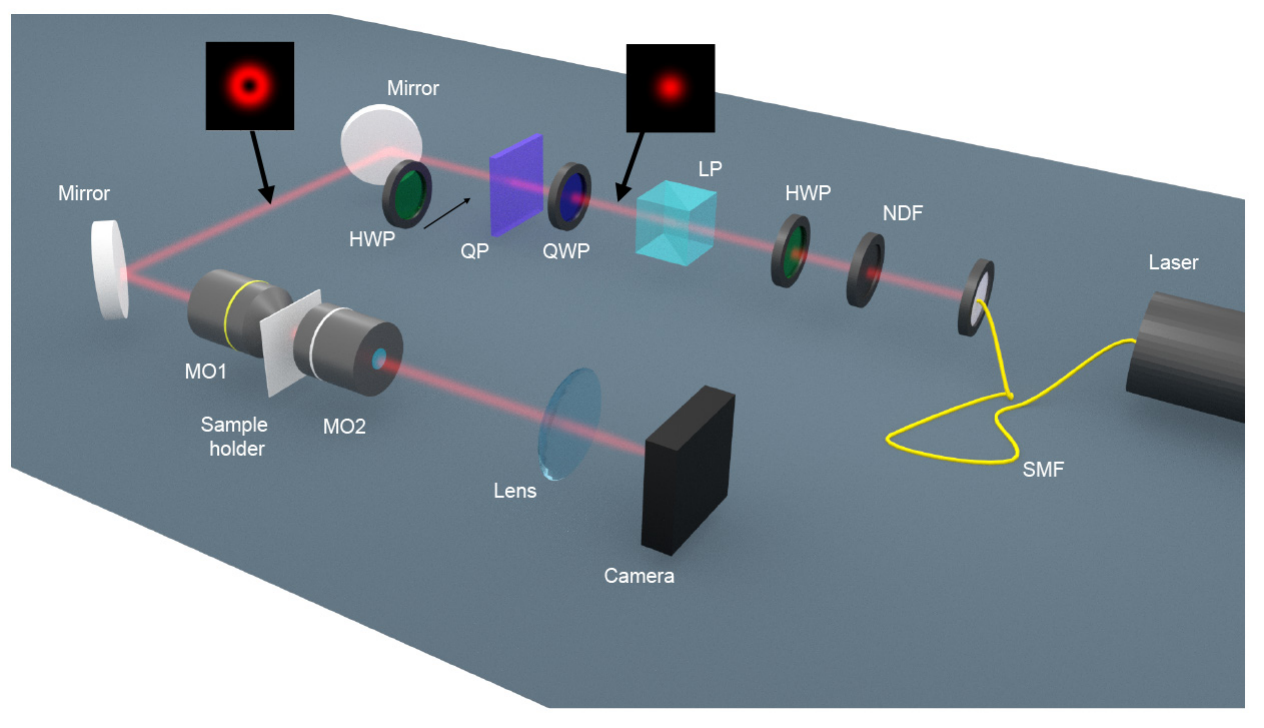
Schematics of the optical set-up that has been used to measure the Vortex Circular Dichroism signal form the cylindrical nanoholes. SMF: single mode fiber. NDF: neutral density filter. HWP: half-wave plate. LP: linear polarizer. QWP: quarter-wave plate. QP: q-plate. MO: microscope objective.

To prove that, we have carried out a set of VCD measurements with cylindrical nanoholes (NH) with diameters ranging from 150 to 1950 nm (0.2 to 3.1 in diameter/wavelength units). Both the LCP and RCP vortex beams that are used for the VCD measurement have a phase singularity of order *l* = −1. The beams have been focused onto the NH with a microscope objective with an NA = 0.9 (MO _1_), and they have been collected with another microscope objective with an NA = 0.8 (MO _2_, see Figure 2). We have observed that the smaller NHs yield VCD signals which are much greater than zero, whereas the larger ones yield a VCD which is approximately zero. Our main claim is that VCD is assessing the vectoriality of this diffraction process, yielding signals similar to zero when the interaction is scalar and signals different from zero when the interaction is vectorial. In order to gain a better understanding of the VCD measurement and confirm our hypothesis, we have also performed some numerical simulations for a different system which shares all the symmetries of our experimental samples but it is easier to simulate: a spherical particle. Using analytical Mie Theory for non-paraxial beams ^8, 12^, we have computed the theoretical VCD for spheres with the same diameters as the NHs used in the experiment. Not only we have obtained very good qualitative agreement with the experimental data, but also we have been able to verify that the VCD signal is related to the degree of polarization-dependence in the diffraction process. Overall, our results show that VCD is a potential technique to experimentally assess the scalar/vectorial regime of a diffraction process.

## Methods

### Optical measurements

The optical set-up in order to perform the VCD measurements is schematically shown in Figure 2. A single mode fiber-coupled HeNe laser ( *λ* = 632.8 nm; Thorlabs HNL210LB) creates a Gaussian beam. The beam goes through a power control system made of a set of neutral density filters (NDF; Thorlabs NDC-100C-2M), a half-wave plate (HWP; Optique J. Fichou, LO-633) and a linear polarizer (LP; Thorlabs GTH10M-A). Then, the polarization is changed to RCP with a quarter-wave plate (QWP: Optique J. Fichou, LO-633). The RCP Gaussian beam then goes through a q-plate ^13^ of order *q* = 1 */*2, which turns the RCP to LCP and adds a spiral phase of the kind *e* ^− *i*φ^. After the q-plate, we eventually place another HWP. When inserted, this HWP changes the handedness of the state from LCP to RCP. In both cases the setup produces a circularly polarized vortex beam with a phase singularity of order *l* = −1. Then, the vortex beam hits the back-aperture of a microscope objective MO _1_ (Olympus, MPLANFL N 100X NA0.9), which has an NA = 0.9. It is important to note here due to the focusing of MO _1_, the polarization at the NH plane (focal plane) is not circularly polarized (see Figure 5). The circular NH sits on top of a sample holder which is attached to a position system composed of a micro and a nanopositioner (PI P-562.3CD). The NHs that are used in this experiment range from 150 nm to 1950 nm, in steps of 100 nm. This range corresponds to 0.2 - 3.1 in diameter/wavelength units. For the smaller NHs, positioning steps of the order of 5 nm need to be taken in order to make sure that the alignment is correct, and that the cylindrical symmetry of the system formed by the sample and beam is maintained. The transmission of the beam through the NH is collected with MO _2_ (Olympus LMPLANFL 100X NA0.8), in this case with an NA = 0.8. The NH lies at the focal plane of MO _2_, therefore the transmission is collimated. The collimated transmission goes through a lens of *f* = 150 mm, which images the transmitted light at the NH plane onto a CMOS camera (The imaging source DMK33UX290). The CMOS camera allows us to measure the differential transmission of the LCP and RCP vortex beams. In order to get a good signal-to-noise ratio for the VCD measurements, we adjust the power of the laser so that the power at the CMOS chip of the camera is at about 80% of the saturation limit. For each of the NH sizes in the 150 - 1950 nm range, we have probed four independent single NHs. All four NHs have been fabricated with the same target diameter. The VCD measurement of each of the four single NHs has been statistically obtained as the result of 80 (4×20) snapshots. We align the NH with respect to the LCP/RCP vortex beam and take 20 snapshots to measure its transmission $I_{l}^{x,t}$ , with *x* = *LCP/RCP*. In order to obtain the $I_{l}^{x,t}$ number, we isolate the area where the beam signal is located in each snapshot, we add the counts from that area, and we subtract the noise signal. This yields $I_{l,i}^{x,t}$ and $I_{l}^{x,t}$ is obtained as the average the 20 $I_{l,i}^{x,t}$ values. Immediately after, we move the sample at 10 *µ*m of distance and take 20 snapshots to measure the transmission of the LCP/RCP through the gold film $I_{l}^{x,in}$ . We compute $I_{l}^{x,in}$ with the same protocol that we use to compute $I_{l}^{x,t}$ . Then, we compute the normalized transmission $I_{l}^{x}=I_{l}^{x,t}/I_{l}^{x,in}$ , and finally the ${\text{VCD}}_{l}=I_{l}^{LCP}-I_{l}^{RCP}$ . Then we divide the VCD _*l*_ result over 10000 so that the result is of the order of 1. Our quick normalization method allows us to correct the power oscillations of the fibre-coupled laser.

### Nanofabrication

**Figure 3.**
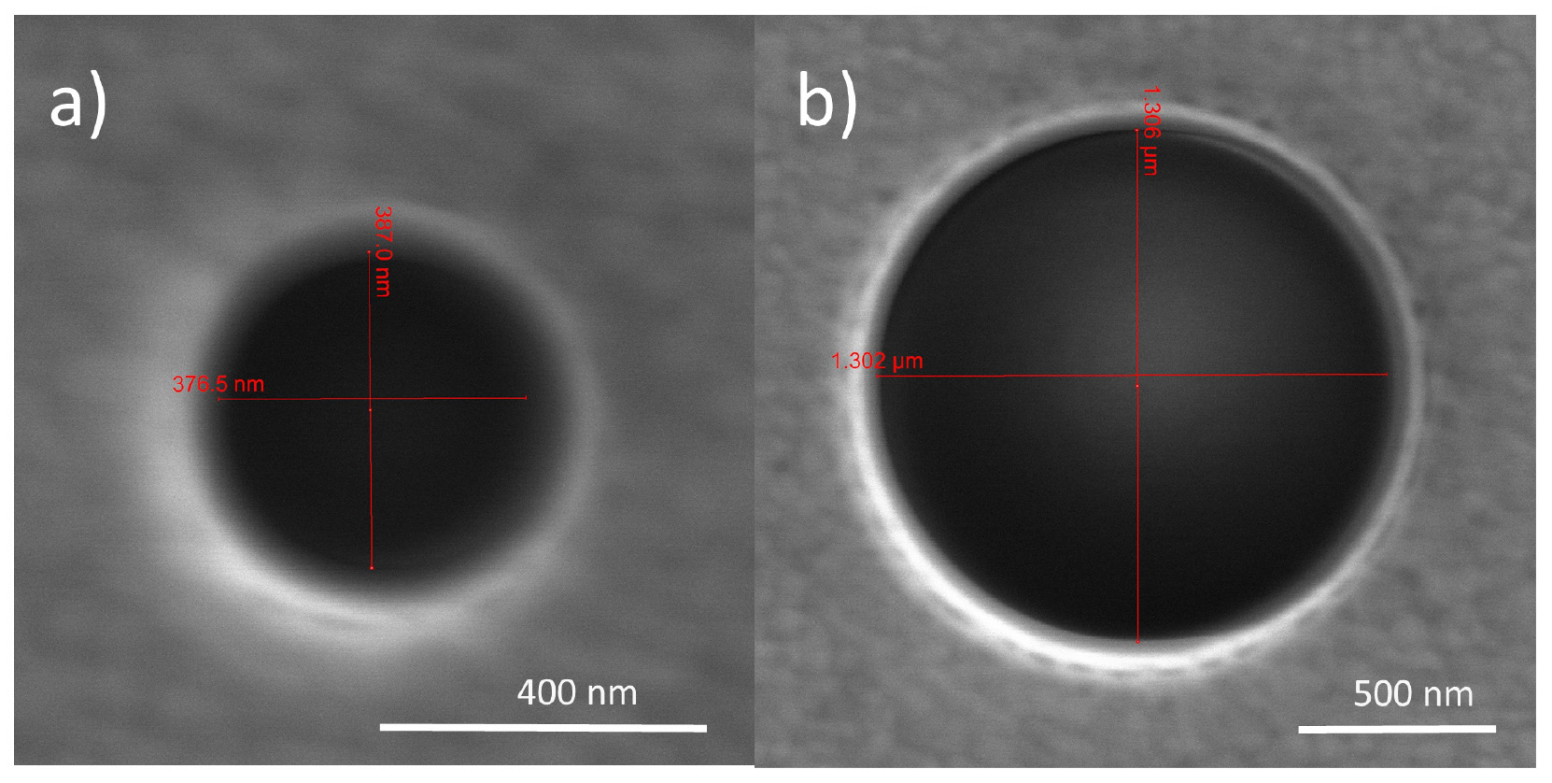
Scanning electron microscope (SEM) images of two nanoholes (NHs), with a diameter of **a**) 380 nm and **b**) 1305 nm.

First, we have deposited a 200 nm gold (Au) layer on top of a 150 *µ*m cover glass. An adhesion layer of 5 nm of titanium (Ti) has been added in between the gold and glass. The deposition of the Au and Ti layers have been carried out by sputtering deposition. Then, the sample composed of the Au, Ti, and Glass layers has been nano-patterned by means of a focused ion beam (FIB). Using the FIB, we have managed to fabricate single circular NHs with diameter sizes ranging from 150 to 1950 nm. Figure 3 shows some of the NHs that have been fabricated. We have fabricated four NHs for each of the target sizes. We have left a distance of at least 25 *µ*m between each NH and its closer neighbors in order to avoid any array effects.

### Numerical simulations

**Figure 4.**
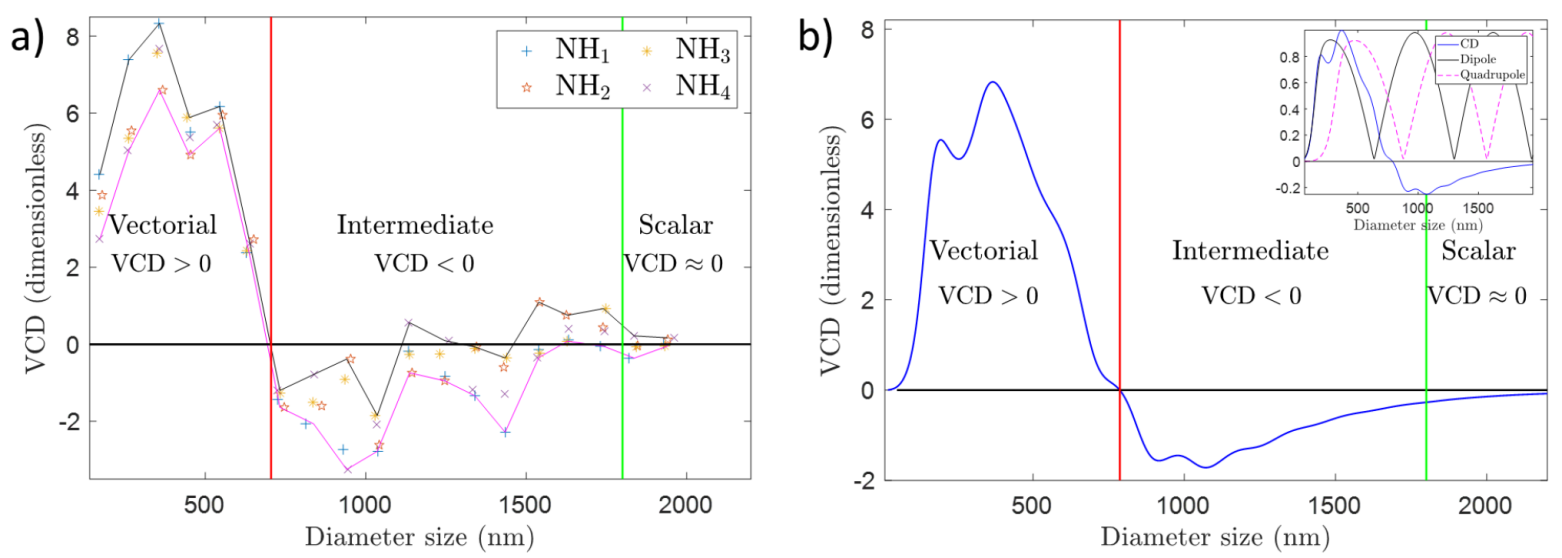
( **a**) Vortex Circular Dichroism (VCD) measurements for a set of nanoholes (NHs) with sizes ranging from 150 to 1950 nm, which correspond to a diameter/wavelength ratio ranging from 0.2 - 3.1 approximately. The laser operates at 632.8 nm. The VCD measurement is defined as the normalized differential transmitted intensity for two vortex beams with opposite circular polarization. The vortex beam that has been used has a phase singularity of order *l* = −1, and it has been focused using a microscope objective with NA= 0.9. The dimensionless units that account for VCD changes are normalized power units (see Methods). ( **b**) Theoretical VCD as a function of the diameter size of a gold particle. The diameter range corresponds to a diameter/wavelength ratio ranging from 0.2 - 3.1 approximately. VCD is computed as $\text{VCD}=\sigma_{abs}^{+}-\sigma_{abs}^{-}.$ The intensity profiles of the incoming beams are given in Figure 5. The wavelength is 632.8 nm. The vortex beam has *l* = −1 and it has been focused with an aplanatic lens with NA= 0.9. The dimensionless units of VCD are explained in Methods. The inset shows the theoretical VCD signal and the dipolar and quadrupolar resonances of the sphere. None of the two resonances peaks at the same position as the VCD signal.

All the numerical simulations included in this manuscript are carried out in the framework of analytical Mie Theory ^14^. The code used to run the simulations is semi-analytical and it is scripted in Matlab. The code solves the classical Mie Theory equations for a non-paraxial vortex beam excitation. An open-source solver for Mie Theory is available on GitHub here. The non-paraxial excitation can be modelled following the equations in ref. 12. The code takes an incident circularly polarized paraxial Laguerre-Gaussian beam ^15^; it focuses it using the aplanatic lens model ^16^ with an NA= 0.9; it decomposes the beam into the multipolar basis ^17^; and then it computes the interaction between this incoming beam and the sphere. As a result, the EM field outside and inside of the sphere is obtained, as well as the scattering and absorption cross sections ^12, 18^. The theoretical VCD is computed by subtracting the absorption cross sections of the LCP and RCP vortex beams used in the experiment. Note that our absorption cross sections *σ _abs_* are dimensionless, and they are computed as the ratio between the absorbed power over the incident power of the beam, which is unitary. Then, the arbitrary units of VCD used in Figure 4b) are the dimensionless cross section units multiplied by a factor of 1000 to make them be of the order of 1. Besides, the scattered EM field is expressed in the basis of $\left\{{\hat{e}}_{+},{\hat{e}}_{-},\hat{z}\right\}$ , where ${\hat{\text{e}}}_{\pm }=\left(\hat{\text{x}}\mp i\hat{\text{y}}\right)/\sqrt{2}$ . Expressing the field in this basis allows us to easily track changes in the polarization of the incident beam.

**Figure 5.**
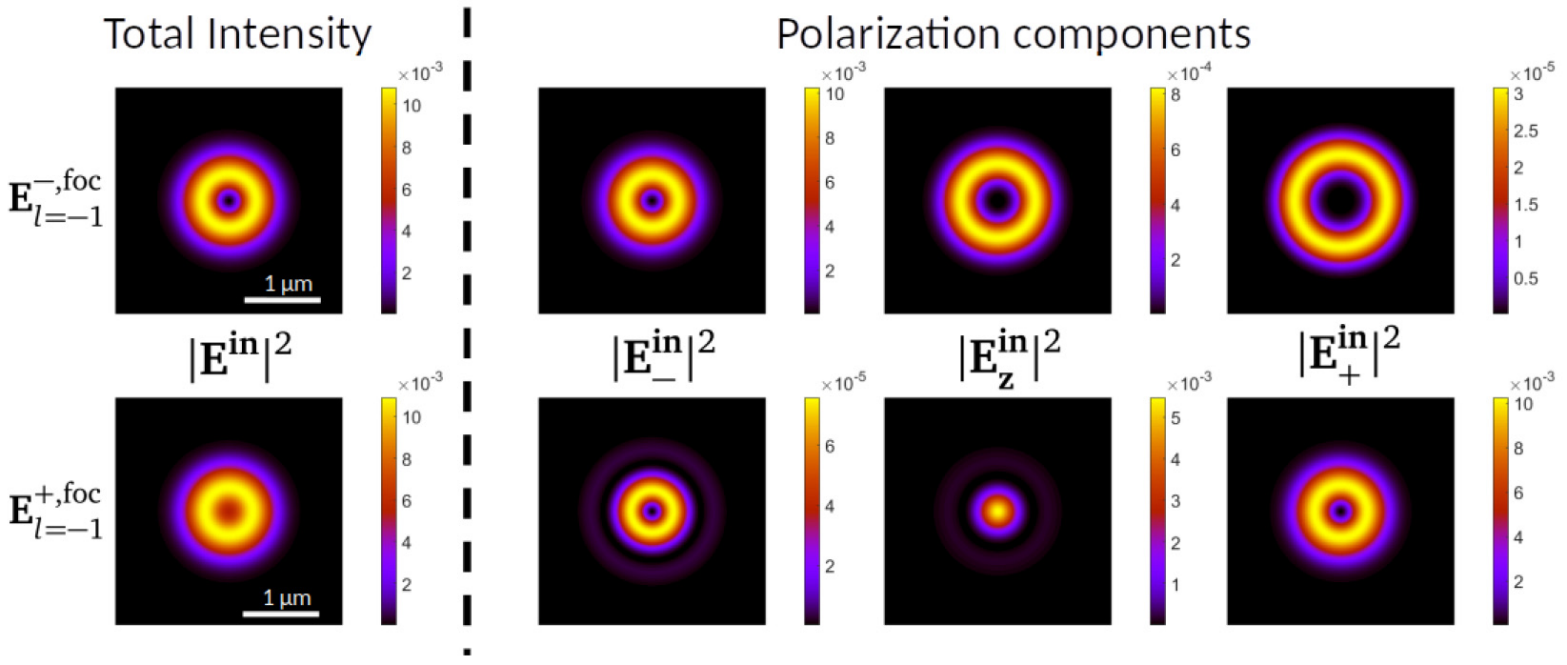
On the left of the dashed vertical line, modulus square of the electric field of the incoming beams $E_{l=-1}^{-,\text{foc}}$ (top) and $E_{l=-1}^{+,\text{foc}}$ (bottom). On the right, projections in the three polarization components $\left\{{\hat{e}}_{-},\hat{z},{\hat{e}}_{+}\right\}$ of $E_{l=-1}^{-,\text{foc}}$ (top) and $E_{l=-1}^{+,\text{foc}}$ (bottom). All the plots are taken at a transverse 4 × 4 *µ*m ^2^ XY plane at the focal plane of the incoming beam. The colorbars show that the differences in intensity between the three polarization components vary across two orders of magnitude. Both $E_{l=-1}^{\pm ,\text{foc}}$ have been obtained applying the aplanatic lens model with an NA=0.9 to a paraxial vortex beam given by equation (2) for *λ* = 632.8 nm.

## Results

The results of our experiments can be observed in Figure 4a), and the full data is available from *Underlying data* ^19^. Figure 4a) shows the variation of VCD as a function of the diameter of the NHs. Notice that describing the VCD measurement as the differential absorption of LCP and RCP vortex beams is an abuse of language, as the two vortex beams are tightly focused, and their polarization at the NH focal plane is not circular (see Figure 5). However, we can use these terms when referring to the polarization state at the rear-aperture of MO _1_. Thus, for the sake of simplicity, we have chosen to extend this notation to the focusing region. It is also important to note that the VCD measurement is performed as a differential power measurement. Because of that, it is of paramount importance to normalize the transmission to the incident power:

$${\text{VCD}}_{l}=I_{l}^{LCP}-I_{l}^{RCP}\;(1)$$

where $I_{l}^{LCP}\left(I_{l}^{RCP}\right)$ is the normalized transmitted power of a LCP (RCP) vortex beam with a phase singularity of order *l*. That is, $I_{l}^{x}=I_{l}^{x,t}/I_{l}^{x,in}$ with *x* = *LC P/RCP*, where $I_{l}^{x,t}$ is the measured transmitted power of LCP/RCP beams through the NHs, and $I_{l}^{x,in}$ is proportional to the value of the power of the incident LCP/RCP beams (see Methods). In our experiment, we have used *l* = −1. As a result, the total angular momentum of the two beams that are used for the VCD measurement is 0 (LCP) and -2 (RCP) ^9, 15, 20, 21^. We have chosen | *l*| = 1 because vortex beams with phase singularities of higher orders are very unstable and they tend to split ^22, 23^, thus making the VCD measurement less reproducible. The sign of *l* has been chosen so that the VCD measurement for small NH sizes was positive. Note that the sign of *l* has a clear impact on the sign of the VCD, as it can be proven that VCD _*l*_ = −VCD _*−l*_^11^. We have carried out VCD measurements for single NHs whose sizes range from 150 to 1950 nm in steps of 100 nm. In diameter/wavelength units this corresponds to 0.2 to 3.1 in size. For each size, we have probed four single NHs. As a result, in Figure 4a), we have plotted four independent data points for each size. Each VCD _*l*_ data point is obtained as a function of 80 snapshots see Methods). The error bars of each individual point in Figure 4a) are not seen as they are smaller than the markers themselves. Moreover, we have also plotted two trend-lines that follow the minima and maxima VCD values for each diameter size to help the eye of the reader. Now, it can be observed that the experimental data shown in Figure 4a) can be split into three different areas. When the diameter of the NHs is approximately below 700 nm (1.1 in diameter/wavelength units), the VCD signal is positive. When the diameter is approximately in between 700 and 1600 nm, VCD yields a negative (but closer to zero) signal. And for diameter sizes greater than 1600 nm (2.5 in diameter/wavelength units), the VCD signal is approximately zero. Figure 4a) is the main experimental result of this work, and it shows that the polarization of a first order non-paraxial vortex beam plays a significant role in the transmission of light through NHs when the size of the NH is below 1600 nm (or 2.5 in diameter/wavelength units). For greater sizes, the polarization does not play any role. That is, the diffraction of our non-paraxial beam through a NH is scalar for sizes above 1600 nm, and it is vectorial for sizes below 700 nm. In between there is an intermediate regime where diffraction is generally vectorial, but it is not far from being scalar - as shown by the crossings of the VCD trend-line with the line VCD = 0. Figure 4a) corroborates what had been sketched in Figure 1: delimiting the vectorial/scalar interaction regime is a difficult endeavor which requires a calculation or a measurement. Our results demonstrate that VCD is a good candidate to quantify the scalar/vectorial regime, since i) it consistently defines the trivial vectorial boundary for NH sizes clearly smaller than *λ*; ii) it captures the subtle vectorial behavior of diffraction in the intermediate cases; iii) it trends towards a scalar behavior for NH sizes larger than *λ*; iv) and it shows a good qualitative agreement with the theoretical VCD reported in Figure 4b). Interestingly, Figure 4a) also shows us that the maximum VCD signal does not happen for the smallest NHs, but rather for some intermediate sizes. Using Mie Theory, we have checked that this is a rather convoluted effect, and it is not directly linked to a single internal resonance of the structure. This can be observed in the inset of Figure 4b), where we have plotted the theoretical VCD signal as well as the dipolar and quadrupolar resonances of a golden sphere. It is seen that none of the two resonances has a maximum at sizes that maximize the VCD signal.

## Discussion

In this section, we carry out some numerical simulations that will help us to strengthen our claims on the validity of VCD as a quantitative technique to determine the scalar/vectorial nature of diffraction. First of all, we have simulated the two focused incoming beams that we use in the VCD measurement, *i.e.* LCP and RCP focused vortex beams with *l* = −1. The analytical expression of their electric field at the rear aperture of MO _1_ can be expressed as:

$${\text{E}}_{l=-1}^{\pm }=A\rho^{-1}L_{0}^{-1}(\frac{2\rho^{2}}{w_{0}^{2}})e^{-\rho^{2/w_{0}^{2}}}e^{i(-\varphi +kz)}{\hat{\text{e}}}_{\pm }\;(2)$$

where *A* is a normalization constant; { *ρ*, *φ*, *z*} are the cylindrical coordinates, with *z* being the propagation axis of the beam; $L_{0}^{-1}$ is a Laguerre polynomial of order *l* = −1; *w*_0_ is the beam waist of the beam at the rear aperture of MO _1_; *k* = 2 *π/λ* is the wavevector, and ${\hat{e}}_{\pm }$ the polarization vector for LCP (RCP) depending on the + (−) sign. Note that the ± sign has to do with the handedness of light, or its helicity ^9, 20^. In order to obtain the electric field at the focal plane of MO _1_, we use the paraxial beam in equation (2) $E_{l=-1}^{\pm }$ and apply the aplanatic lens model ^16^, yielding $E_{l=-1}^{\pm ,\text{foc}}$ . In Figure 5, we display $|E_{l=-1}^{\pm ,\text{foc}}{|}^{2}$ , that is, the modulus square (intensity) of the electric field distribution of $E_{l=-1}^{\pm }$ at the focal plane of a lens with NA = 0.9 for a *λ* = 632.8 nm. The top and bottom rows display the intensity of the $E_{l=-1}^{-,\text{foc}}$ and $E_{l=-1}^{+,\text{foc}}$ beams respectively. Each row is composed of four images, corresponding to the total intensity, and its projections into the three polarization components **ê**_–_, **ê**_*z*_, **ê**_+_. We observe that the two beams have a different intensity profile, where $E_{l=-1}^{+,\text{foc}}$ concentrates more energy around the center of symmetry due to the Gaussian-like $\hat{z}$ component ^9, 11, 20^. Now, given these two incoming focused vortex beams, we simulate the VCD that they induce on spherical golden particles. To do that, we decompose the incoming beams into multipoles using the method put forward in Refs. 12, 15 and solve the analytical Mie Theory problem. As a result, we obtain the scattered EM field **E ^s^** as well as the scattering and absorption cross sections ^14^. We compute the theoretical VCD as:

$$\text{VCD}=\sigma_{abs}^{+}-\sigma_{abs}^{-}\;(1)$$

where both $\sigma_{abs}^{\pm }$ are the absorption cross sections obtained with a focused vortex beam with *l* = −1, and where the +(−) sign has to do with a LCP (RCP) before the back-aperture of the focusing lens.

In Figure 4b), we plot the theoretical VCD obtained with the two beams plotted in Figure 5 as a function of the sphere diameter. Note that we normalize the plot in a way such that the values that we obtain are in the order of 1 (see Methods). We observe that we recover most of the features of Figure 4a). We see that the VCD yields a positive result for the smaller diameters. We also observe that there is an intermediate zone where VCD yields negative (yet closer to zero) values. The scalar zone of VCD ≈ 0 for greater particle sizes is also present in Figure 4b). Besides, we also see that the relative variations in VCD values (in between -2 and 6 given our normalization, see Methods) are comparable to those observed in Figure 4a), despite the fact that Figure 4b) is carried out for a single sphere, which clearly is a different system from a single NH despite sharing some symmetries (mirror and cylindrical). Finally, notice that changes in the geometry, material or NA, would certainly have an effect in the experimental and theoretical VCD trend line. Yet, the comparison between Figures 4a) and 4b) still leads us to believe that VCD has a good potential to probe the vectoriality of diffraction processes.

**Figure 6.**
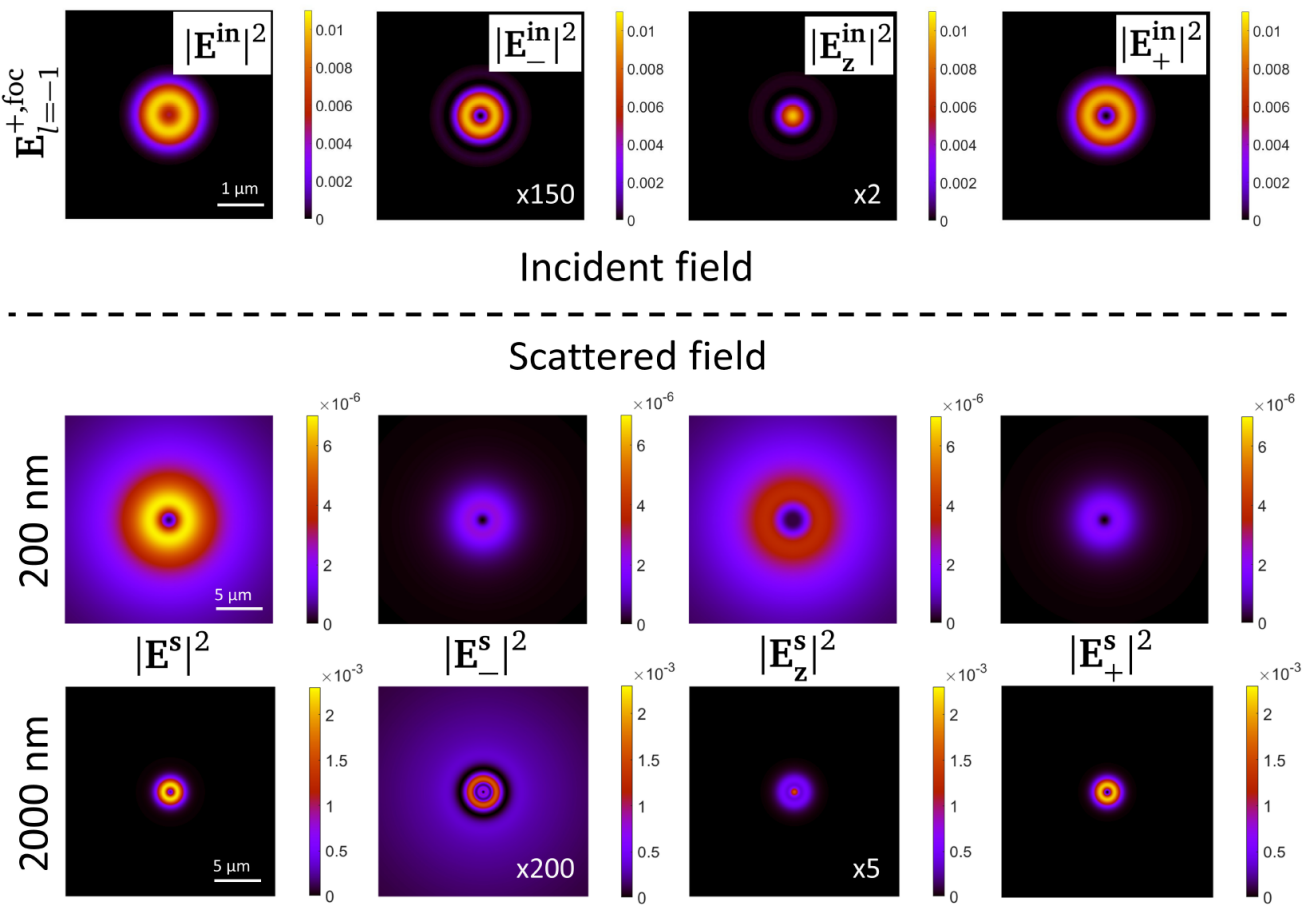
Modulus square of the scattered field **E ^s^** as well as its projection in the three polarization components $\left\{{\hat{e}}_{-},\hat{z},{\hat{e}}_{+}\right\}$ for two spheres with diameters equal to 200 nm (middle row) and 2000 nm (bottom row). The incident field inducing the scattered field is $E_{l=-1}^{+,\text{foc}}$ , and its intensity profile as well as its polarization components at the focal plane are plotted in the top row of the image. The scattering plots are taken at a transverse 40 × 40 *µ*m ^2^ XY plane at distance of *z* = 2 *µ*m of the center of the sphere. The colorbars of all the plots in one row are the same. The intensity of some of the polarization components has been multiplied times a factor. The factor has been written in the plot as ×5.

In order to further support this claim, in Figure 6 we plot the modulus square of the scattered field off the sphere at a transverse XY plane, at a distance *z* = 2 *µ*m from the center of the sphere. We use $E_{l=-1}^{+,\text{foc}}$ as the incoming beam, which is plotted as the top row in Figure 6. A scalar diffraction process is characterized by a negligible role of the polarization of light. In other words, a scalar diffraction process will not change the polarization state of an incoming beam, because the interaction is polarization-independent. The bottom row of Figure 6 shows exactly that: the intensity pattern as well as the polarization projections of the scattered field off a sphere of 2000 nm of diameter are almost the same as the incident beam $E_{l=-1}^{+,\text{foc}}$ (see Figure 6, top row). That is, most of the intensity comes from the **ê**_+_ polarization component, whereas the $\left\{{\hat{e}}_{-},\hat{z}\right\}$ components are negligible. And indeed, this corresponds to a case of VCD ≈ 0. In contrast, the middle row of Figure 6 shows exactly the opposite, *i.e.* a sphere with a diameter of 200 nm strongly changes the polarization of the incident beam. In this case, indeed the scattered field is not dominated by a single polarization component, instead the three $\left\{{\hat{e}}_{-},\hat{z},{\hat{e}}_{+}\right\}$ polarization components yield a similar intensity (see the colorbars in Figure 6). This is a consequence of the fact that the interaction is vectorial, and therefore it is not polarization-independent. And as expected, VCD ≠ 0.

Overall, Figure 6 supports the main claim of this article, namely that VCD is an experimental technique that allow us to assess the scalar/vectorial regime of a diffraction process.

## Conclusions

Vortex Circular Dichroism (VCD) is an experimental technique that consists in carrying out a circular dichroism (CD) measurement with a vortex beam. We have presented some experimental data and theoretical simulations to support the following three claims. i) The vectoriality of a diffraction process can be experimentally assessed. ii) The transition between the scalar and vectorial regimes in a diffraction process is subtle and not easy to predict, hence its characterization calls for a measuring technique. iii) VCD is a potential candidate to assess the scalar/vectorial regime of a diffraction process. The experimental data has been collected for single nanoholes in a gold film, while the theoretical calculations have been done for golden spheres. Despite having different geometries, both systems have yielded a very similar trend-line of VCD vs size of the nanostructure (see Figure 4): VCD > 0 for small sizes, VCD ≲ 0 for intermediate sizes and VCD ≈ 0 for larger sizes. Some extra simulations of the scattered far-field (see Figure 6) have corroborated that scalar (vectorial) diffraction processes in which VCD ≈ 0 (VCD ≠ 0) are (not) polarization-independent. In our experiment we considered a diffraction process involving a first order vortex beam. Note that if the measurement was done with a Gaussian beam, no information about the scalar/vectorial regime would be found, as the CD measurement would be 0 for all the sizes. This is due to the fact that a LCP and RCP Gaussian beam are mirror symmetric, whereas the LCP and RCP vortex beams used in VCD are not ^9, 11^. Overall, our work shows that VCD has all the necessary features to be a measure of vectoriality of a diffraction process involving non mirror-symmetric beam pairs such as, for instance, the well known radial and azimuthal polarized beams ^24, 25^. As it happens with most of new measuring techniques, its direct applications are still to be thought of. Yet it is clear that the experimental quantification of the scalar/vectorial regime could contribute to gain a more in-depth knowledge of fundamental light-matter interactions. In particular, it could be an important asset in order to program experiments and/or simulations in an optimized way, *e.g*. saving a vectorial simulation and running a scalar one instead.

## Ethics and consent

Ethical approval and consent were not required.

## Data Availability

Zenodo: Vortex Circular Dichroism experiment at IIT. https://doi.org/10.5281/zenodo.7319991 ^19^. This project contains the following underlying data, sorted in three different files: *Snapshots.zip*. This compressed file contains all the snapshots that have been taken in order to carry out the VCD measurements. As stated in *Methods*, there are four categories of snapshots concerning the transmission of vortex beams under four different conditions: 1) Transmission of LCP vortex beams through the nanohole; 2) Transmission of RCP vortex beams through the nanohole; 3) Transmission of LCP vortex beams through the gold layer; 4) Transmission of RCP vortex beams through the gold layer. The notation of the files is (n)h[size in nm]_[pol]_[pos]_[num].tiff, where h (nh) is written when the light passes (does not pass) through the nanohole. [size in nm] gives us the diameter of the nanohole in nm. [pol] tells us if the incident beam is LCP ( [pol]=60) or RCP ( [pol]=0). [pos]=1,2,3,4 refers to the four nanoholes that have been probed for each diameter size. [num] is the snapshot number, as 10 snapshots were taken for each nanohole. *Data.ods*. This file contains two sets of data, sorted in two different tabs. In the *Conversion Table* tab, we find a table that allows us to give units to the snapshots given in the *Snapshots.zip* file. The table is needed because in the measurements we regulated the power of the laser to almost saturate the CMOS chip of the camera. Moreover, the same table can be later used to turn the VCD measurement dimensionless. Then, in the *Nanoholes sizes* tab, we use the snapshots collected in the *SEM_nanoholes.zip* file to retrieve the sizes of the nanoholes that have been used in the experiment. *SEM_nanoholes.zip*. This file contains all the SEM measurements (.jpeg) to retrieve the diameter of the nanoholes used in the experiment. Data are available under the terms of the Creative Commons Attribution 4.0 International license (CC-BY 4.0).

## References

[1] Saleh BEA Teich MC : Fundamentals of photonics. Wiley New York,1991;22. 10.1002/0471213748

[2] Jackson JD : Classical Electrodynamics: Third Edition. *Am J Phys.* John Wiley & Sons, New York,1999;67( *9*):841. 10.1119/1.19136

[3] Gupta SD Ghosh N Banerjee A : Wave optics: Basic concepts and contemporary trends. CRC sress,2015. 10.1201/b19330

[4] Sirohi RS : Wave optics and its applications. Orient Blackswan,1993. Reference Source

[5] Lax M Louisell WH McKnight WB : From maxwell to paraxial wave optics. *Phys Rev A.* 1975;11(4):1365. 10.1103/PhysRevA.11.1365

[6] Vaveliuk P Ruiz B Lencina A : Limits of the paraxial approximation in laser beams. *Opt Lett.* 2007;32(8):927–929. 10.1364/ol.32.000927 17375156

[7] Mie G : Beiträge zur optik trüber medien, speziell kolloidaler metallösungen. *Annalen der Physik.* 1908;330(3):377–445. 10.1002/andp.19083300302

[8] Gouesbet G Gréhan G : Generalized Lorenz-Mie Theories. Springer,2011. 10.1007/978-3-642-17194-9

[9] Zambrana-Puyalto X Vidal X Fernandez-Corbaton I : Far-field measurements of vortex beams interacting with nanoholes. *Sci Rep.* 2016;6:22185. 10.1038/srep22185 26911547 PMC4766500

[10] Barron LD : Molecular light scattering and optical activity. Cambridge University Press,2009. 10.1017/CBO9780511535468

[11] Zambrana-Puyalto X Vidal X Molina-Terriza G : Angular momentum-induced circular dichroism in non-chiral nanostructures. *Nat Commun.* 2014;5:4922. 10.1038/ncomms5922 25215603

[12] Zambrana-Puyalto X Vidal X Molina-Terriza G : Excitation of single multipolar modes with engineered cylindrically symmetric fields. *Opt Express.* 2012;20(22):24536–24544. 10.1364/OE.20.024536 23187217

[13] Marrucci L Manzo C Paparo D : Optical spin-to-orbital angular momentum conversion in inhomogeneous anisotropic media. *Phys Rev Lett.* 2006;96(16):163905. 10.1103/PhysRevLett.96.163905 16712234

[14] Bohren CF Huffman DR : Absorption and scattering of light by small particles. Wiley,1983. Reference Source

[15] Zambrana-Puyalto X : Control and characterization of nano-structures with the symmetries of light. PhD thesis, Macquarie University,2014. Reference Source

[16] Novotny L Hecht B : Principles of Nano-Optics. Cambridge University Press,2006. 10.1017/CBO9780511813535

[17] Rose ME : Elementary Theory of Angular Momentum. Wiley, New York,1957. Reference Source

[18] Zambrana-Puyalto X Molina-Terriza G : The role of the angular momentum of light in mie scattering. excitation of dielectric spheres with laguerre-gaussian modes. *J Quant Spectrosc Radiat Transf.* 2013;126:50–55. 10.1016/j.jqsrt.2012.10.010

[19] Zambrana-Puyalto X : Vortex Circular Dichroism experiment at IIT. [Data set]. Zenodo. 2022. 10.5281/zenodo.7319991

[20] Fernandez-Corbaton I Zambrana-Puyalto X Molina-Terriza G : Helicity and angular momen-tum: A symmetry-based framework for the study of light-matter interactions. *Phys Rev A.* 2012;86:042103. 10.1103/PhysRevA.86.042103

[21] Zambrana-Puyalto X Vidal X Woźniak P : Tailoring multipolar mie scattering with helicity and angular momentum. *ACS Photonics.* 2018;5(7):2936–2944. 10.1021/acsphotonics.8b00268

[22] Ricci F Löffler W Van Exter MP : Instability of higher-order optical vortices analyzed with a multi-pinhole interferometer. *Opt Express.* 2012;20(20):22961–22975. 10.1364/OE.20.022961 23037446

[23] Neo R Tan SJ Zambrana-Puyalto X : Correcting vortex splitting in higher order vortex beams. *Opt Express.* 2014;22(8):9920–9931. 10.1364/OE.22.009920 24787874

[24] Banzer P Kindler J Quabis S : Extraordinary transmission through a single coaxial aperture in a thin metal film. *Opt Express.* 2010;18(10):10896–10904. 10.1364/OE.18.010896 20588945

[25] Woźniak P Banzer P Leuchs G : Selective switching of individual multipole resonances in single dielectric nanoparticles. *Laser Photonics Rev.* 2015;9(2):231–240. 10.1002/lpor.201400188

