# Supplementary material for: Vortex Circular Dichroism: An experimental technique to assess the scalar/vectorial regime of diffraction
Source: Open Res Eur. 2024 May 22;2:142. Originally published 2022 Dec 19. [Version 2] doi: 10.12688/openreseurope.14916.2 (PMC11140307; doi:10.12688/openreseurope.14916.2)
Supplement: Peer review history

# Reviewer report: Reviewer response for version 2

**DOI:** 10.21956/openreseurope.18803.r40799

**Published:** 2024-05-30

**Authors:** Andrew Forbes

**Affiliations:** 1 School of Physics, University of the Witwatersrand, Johannesburg, South Africa

No further comments to make.

Is the study design appropriate and does the work have academic merit?

Yes

Is the work clearly and accurately presented and does it cite the current literature?

Yes

If applicable, is the statistical analysis and its interpretation appropriate?

No

Are all the source data underlying the results available to ensure full reproducibility?

Yes

Are the conclusions drawn adequately supported by the results?

Yes

Are sufficient details of methods and analysis provided to allow replication by others?

Yes

Reviewer Expertise:

structured light

I confirm that I have read this submission and believe that I have an appropriate level of expertise to confirm that it is of an acceptable scientific standard.

# Reviewer report: Reviewer response for version 1

**DOI:** 10.21956/openreseurope.16119.r31133

**Published:** 2023-05-12

**Authors:** Andrew Forbes

**Affiliations:** 1 School of Physics, University of the Witwatersrand, Johannesburg, South Africa

The authors question the onset of regimes where vectorial light treatment is needed and where scalar is ok. To do this they pass light through nanoholes of various sizes in relation to the wavelength and look at the effect on CD as a test. Everything looks solid except for the generalised conclusions. Finding a trend for THIS particular system and something similar in one other system does not imply that it is universally true. As far as I could see, each measurement was performed only once, so I doubt one can make sweeping claims. Also, the difference between CD and VCD was buried in the text and could sure be brought our more (mathematically) since it is central to the story. On a minor note, the technical terms are often a little odd, e.g., unitary has a specific meaning and is not usually used to describe a unit vector, and singular beams with a phase singularity and vortex beams (carrying OAM) are not the same thing - see recent papers by Michael Berry on the topic.

 Having said this, I do like the simplicity of the question and its experimental test, so just a few adjustments to the main claims or at least a discussion for the reader to consider arguments would be in order.

Is the study design appropriate and does the work have academic merit?

Yes

Is the work clearly and accurately presented and does it cite the current literature?

Yes

If applicable, is the statistical analysis and its interpretation appropriate?

No

Are all the source data underlying the results available to ensure full reproducibility?

Yes

Are the conclusions drawn adequately supported by the results?

Yes

Are sufficient details of methods and analysis provided to allow replication by others?

Yes

Reviewer Expertise:

structured light

I confirm that I have read this submission and believe that I have an appropriate level of expertise to confirm that it is of an acceptable scientific standard, however I have significant reservations, as outlined above.

# Response

**Published:** 2024-04-29

**Authors:** Xavier Zambrana-Puyalto

**Affiliations:** Istituto Italiano di Tecnologia, Italy

We thank Andrew Forbes for reviewing our article. His comments have definitely helped us to improve our work. Next, we address his points, one by one. To see a more complete response with figures, equations and references, see this PDF file attached here.

  

- We thank the referee for pointing this out. We tried to be careful, as we never wanted to imply that VCD is a universal measurement of the vectoriality of diffraction. We think that it is rather a technique that works for the cases that we have studied. Due to its features, we envision that its use could be extended to other cases, but that needs to be demonstrated, and this was not the aim of this work. As a result, we have changed several sentences in the manuscript to make this clear. For example, we have changed the last sentence of the abstract to: "Overall, our results show compelling evidence that it is possible to experimentally assess the scalar/vectorial regime of a diffraction process, and that the VCD technique possesses the required features to measure the vectoriality of diffraction processes involving plasmonic cylindrically symmetric structures."

We thank the referee for the comment about the differences between the CD and VCD measurements. It is indeed central to the story, and it needs to be highlighted. In the revised version, we have included the following sentences:

- "In this work [11], it was shown that there are two main differences between a standard CD measurement using Gaussian beams or plane waves, and the CD measurement using vortex beams: i) The CD measurement is a differential measurement between two beams that are connected via a mirror symmetry. That is, a LCP Gaussian beam and a RCP Gaussian beam are mirror images with respect to each other. In contrast, the VCD measurement uses a LCP and a RCP vortex beam, and it can be demonstrated that these two beams are not mirror symmetric images - the spiral phase of the vortex beam breaks this symmetry. ii) The two Gaussian beams (LCP and RCP) used in the CD measurement have an angular momentum content of 1 unit, whereas the angular momentum content of the two vortex beams used for the VCD experiment have a different angular momentum content equal to 0 and 2 units. Both i) and ii) are symmetry considerations, and they show why CD must be zero under normal incidence, and why VCD does not need to be zero. Yet, these symmetries consideration do not explain the physical mechanism that creates a non-zero VCD measurement for certain samples." On the minor note - we have changed the expression unitary vector to unit vector. We have also cited two of the latest works of Michael Berry and written the following paragraph, so that the readers know that we interchangeably write vortex beam and beam with phase singularity to mean paraxial beams which are eigenstates of the OAM operator. "Note that in general, vortex beams carrying angular momentum and beams with phase singularities are not equivalent [12,13]. But in this work, we use the terms vortex beam and beam carrying a phase singularity interchangeably because we use vortex beams with a single phase singularity of the kind exp(-i*phi).

# Reviewer report: Reviewer response for version 1

**DOI:** 10.21956/openreseurope.16119.r31135

**Published:** 2023-04-26

**Authors:** Alejandro Martínez

**Affiliations:** 1 Nanophotonics Technology Center, Universitat Politècnica de València, Valencia, Spain

Essentially, the authors use the technique unveiled in Ref. 11 to study the vectorial nature of diffraction when light impinges on objects (holes experimentally, spheres theoretically) of different sizes. Now the experiments go further to explain the surprising existence of non-zero VCD (requiring light with non-zero orbital angular momentum) on non-chiral samples when light-matter interaction is vectorial (small scatterers). The authors observe that VCD is nonzero for objects of size comparable to or smaller than the wavelength, a behavior that they attribute to the measure of vectoriality of a diffraction process. Numerical results using Mie theory also support this finding and, surprisingly, they find very similar regimes of performance (see Figs. 4a and b) in experiments and theory even though employing very different scatterers (holes vs. spheres). I think that these results are of interest to scientists doing research in light-matter interaction, in particular those working with polarized and structured light at the nanoscale. As so I would recommend indexing. Still, there are some doubts that come to my mind after reading the manuscript:

- My main concern is why VCD starts being positive (for very small scatterers) and then its sign switch to negative before tending to zero for large objects. Any explanation for this phenomenon that is consistently observed in holes and spheres?
- Does spin-orbit interaction plays any role in the whole picture? For focused beams being circularly polarized, interaction with small objects should be affected by this interaction.
- The Au layer is quite thick (200 nm). Would the results be the same using thinner Au layers (as conventionally done in plasmonics)? Does the thickness play any role?
- This sentence may be misleading: “On the one hand, the scalar regime is suitable to describe diffraction processes where the dimensions of both the beams and matter are much greater than the wavelength” since one usually relates diffraction with light illuminating objects with sizes of the order of (or smaller than) the wavelength.

Is the study design appropriate and does the work have academic merit?

Yes

Is the work clearly and accurately presented and does it cite the current literature?

Yes

If applicable, is the statistical analysis and its interpretation appropriate?

Yes

Are all the source data underlying the results available to ensure full reproducibility?

Yes

Are the conclusions drawn adequately supported by the results?

Yes

Are sufficient details of methods and analysis provided to allow replication by others?

Yes

Reviewer Expertise:

Nanophotonics

I confirm that I have read this submission and believe that I have an appropriate level of expertise to confirm that it is of an acceptable scientific standard.

# Response

**Published:** 2024-04-29

**Authors:** Xavier Zambrana-Puyalto

**Affiliations:** Istituto Italiano di Tecnologia, Italy

We thank Alejandro Martínez for reviewing our article. His comments have definitely helped us to improve our work. Next, we address his points, one by one. To see a more complete response with figures, equations and references, see this PDF file attached here.

  

- Intuitively, we think that this effect is a consequence of the differences in beam waist of the two beams used to perform the VCD measurement. If we compare the field structure at the focus of the two different beams used for the VCD measurement, a beam with a total AM equal to 0 and another one with AM equal to 2, we observe that the field at the focus has a smaller beam waist (this can be observed in Figure 5 of the manuscript). Now, for Mie particles, we know that we usually couple more light into the resonator when the incident beam is tangential to the surface. As a result, the beam with AM=0 in-couples more light for particles with diameters below 600 nm, and the beam with AM=2 in-couples more light with diameters between 900 and 1600 nm. The z polarization component of the incident beams with l=-1 is particularly revealing for this consideration. This is shown in the figure of the pdf, where we show the z component of the two incident beams and we compare them with a circumference of diameter = 500 nm (top row) and diameter = 1200 nm (bottom row). For D = 500 nm, we observe that the beam with helicity=-1 illuminates the particle more tangentially (or via surface-scattering) than the beam with helicity=+1, whereas for D = 1200 nm is the other way around.
- We can describe light matter-interactions with different frameworks. For instance, one can describe the interaction between non-paraxial beams of light and nano-structures using the spin and orbital angular momentum. This description has been used by many authors in the field. Alternatively, one can also choose to describe the problem in terms of the total angular momentum and the handedness (or helicity) of light beams. This is what we have decided to do here, as it is a description that we are more familiar with and that we believe that it is better suited for these sort of problems. In the revised manuscript, we have included the following sentence that reminds the readers that the problem could have been addressed within the spin-orbit interactions framework: "Notice that our theoretical modelling is based on Mie Theory and a beam description based on the total angular momentum and handedness, yet some other authors have addressed this problem in the framework of spin-orbit interactions [25-27]."
- The 200 nm layer of gold is used to make sure there is no direct transmission of the beam through the gold layer. As long as the thickness of the Au layer is such that there is no direct transmission of the laser beam, we do not foresee any big changes in the VCR(d) trend-line. However, a drastic reduction of the thickness of the layer of gold could turn the Au-glass system into a multilayer system and the VCR(d) trend-line would be affected as a result. Even if the effect of the thickness is not in the scope of this work, we have added a sentence in the main article to mention the effect of the thickness: "Note that the thickness of the Au layer has been chosen to greatly reduce the direct transmission of the beams through the sample. It is not expected that slight changes in the thickness of the gold layer will modify the results of this paper."
- We thank the referee for pointing this out. We agree that the sentence was confusing, and therefore we have changed it. Now, it reads as:
  "On the one hand, the scalar regime is suitable to describe diffraction processes where *either* the beam or the matter size are much greater than the wavelength.
